# Evaluation of Minnesota Score in the Allocation of Venovenous Extracorporeal Membrane Oxygenation During Resource Scarcity

**DOI:** 10.1155/2022/2773980

**Published:** 2022-04-06

**Authors:** Jillian K. Wothe, Zachary R. Bergman, Arianna E. Lofrano, Melissa Doucette, Ramiro Saavedra-Romero, Matthew E. Prekker, Elizabeth R. Lusczek, Melissa E. Brunsvold

**Affiliations:** ^1^Medical School, University of Minnesota, Minneapolis, MN, USA; ^2^Department of Surgery, University of Minnesota, Minneapolis, MN, USA; ^3^Department of Internal Medicine, Hennepin Healthcare, Minneapolis, MN, USA; ^4^Department of Critical Care Medicine, Abbott Northwestern Hospital, Minneapolis, MN, USA; ^5^Department of Emergency Medicine, Hennepin Healthcare, Minneapolis, MN, USA

## Abstract

**Background:**

In this study, we evaluate the previously reported novel Minnesota Score for association with in-hospital mortality and allocation of venovenous extracorporeal membrane oxygenation in patients with acute respiratory distress syndrome with or without SARS-CoV-2 pneumonia.

**Methods:**

This was a retrospective cohort study across four extracorporeal membrane oxygenation centers in Minnesota. Logistic regression was used to assess the relationship between the scores and in-hospital mortality, duration of ECMO cannulation, and discharge disposition. Priority groups were established statistically by maximizing the sum of sensitivity and specificity and compared to the previous qualitatively established priority groups.

**Results:**

Of 124 patients included in the study, 38% were treated for COVID-19 acute respiratory distress syndrome. The median age was 48 years, and 73% were male. The in-hospital mortality rate was 38%. The Minnesota Score was significantly associated with in-hospital mortality only (OR 1.13, *p*=0.02). Statistically determined cut points were similar to qualitative cut points. SARS-CoV-2 status did not change the findings.

**Conclusions:**

In our patient cohort, the Minnesota Score is associated with increased mortality. With further validation, proposed priority groups could be utilized for allocation of ECMO in times of increasing scarcity.

## 1. Introduction

Venovenous extracorporeal membrane oxygenation (V-V ECMO) improves survival of patients with severe respiratory failure, particularly in those with acute respiratory syndrome (ARDS) [1]. SARS-CoV-2 (COVID-19) appeared in late 2019 and is associated with a range of clinical courses [2]. In severe cases, pneumonia from COVID-19 can cause ARDS, leading to poor outcomes [3, 4]. V-V ECMO has been successfully used in the treatment of these patients with recent studies reporting survival rates similar to pre-COVID-19 patients. [5–7].

Shortages of equipment and personnel have been reported throughout the pandemic in part due to the lack of an organized, national response [8]. ECMO requires extensive resources and patients with ARDS secondary to COVID-19 pneumonia tend to have long runs, with some studies reporting averages as high as 17–21 days [5, 6, 9]. This can exacerbate shortages and has led to a need for a system for allocation of ECMO that fairly and accurately assesses a patient's likelihood of benefit during periods of scarcity. The RESP score, which is often used to predict survival for patients treated with ECMO for respiratory failure is neither validated in patients with COVID-19 nor was designed as an allocation tool during times of scarcity [10]. In March 2020, the Extracorporeal Life Support Organization (ELSO) released guidelines for use of V-V ECMO during the COVID-19 pandemic which prioritized younger patients without significant comorbidities [11]. Various private and public health groups have attempted to expand on this recommendation with more elaborate algorithms [12]. While several allocation systems have been proposed, to our knowledge, their performance has not been formally assessed.

Early in the pandemic, the Minnesota ECMO Consortium, which consists of four ECMO directors in the state of Minnesota, worked together to formulate an allocation system to use at their centers. This led to the creation of the Minnesota (MN) Score, which is described in a previous paper by our group and was adapted from a previously published model for allocation of critical care resources by White et al. [12, 13]. The qualitatively derived MN Score was intended to be used to help determine allocation of ECMO in the state should resources become scarce. In this paper, we statistically evaluate the MN Score in patients treated with V-V ECMO for ARDS before and during the pandemic to assess its utility for resource allocation and its association with key outcomes of mortality, duration of ECMO cannulation, and discharge destination. We also compare it to the RESP score.

## 2. Materials and Methods

### 2.1. Study Design and Participants

**Figure 1:**
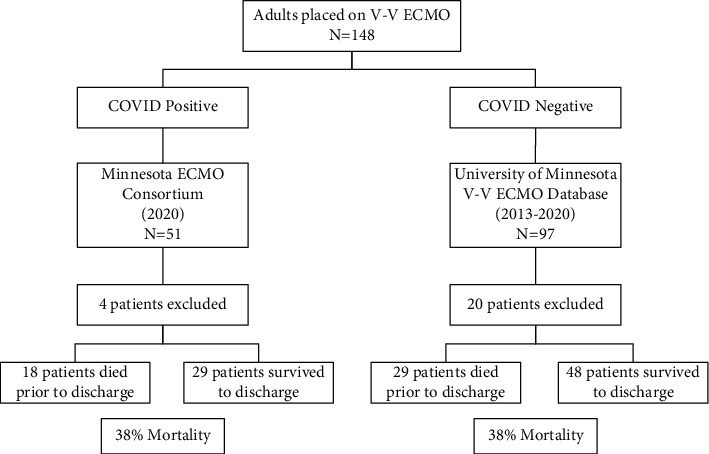
Flow chart describing patient selection. There were two primary groups that were evaluated. COVID-positive patients were treated at one of four ECMO centers of excellence in 2020. COVID-negative patients were treated at the University of Minnesota from 2013 to 2020. Mortality was equal between the groups (*p*=0.94).

This was a retrospective study that included patients placed on V-V ECMO for ARDS who were treated at one of four adult Extracorporeal Life Support Centers of Excellence in Minnesota from 2013 to 2020. The populations are summarized in Figure 1, which included a subanalysis to evaluate the effect of COVID-19. The first population included adult patients receiving V-V ECMO for ARDS at the University of Minnesota from January 1, 2013, to December 31, 2020, excluding patients who received ECMO as a bridge to lung or heart transplantation or who received ECMO as well as an implantable ventricular assist device for refractory heart failure. The second population included patients with ARDS due to COVID-19 pneumonia who received V-V ECMO at one of the four adult ECMO centers in Minnesota. Using retrospective chart review, we collected patient and disease characteristics as well as outcomes including in-hospital mortality, ECMO duration, and discharge disposition. We then calculated each patient's MN Score and RESP score using data prior to the time of cannulation. All data was stored in a REDCap electronic data capture tool provided by the University of Minnesota [14]. The study was reviewed and approved by the institutional review board at each institution.

### 2.2. Minnesota Score

The MN Score consists of three domains that are superimposed on a matrix to produce a single score ranging from 3 to 22. The first domain is a 3 × 2 matrix that integrates anticipated survival and duration of ECMO based on indication for V-A or V-V ECMO. The second domain is the SOFA score and has four groups: less than 6, 6–8, and 9–11, and greater than or equal to 12. The third domain is age and has 3 groups: less than 40, 41–60, and 61–75. The final score is derived from a matrix that assigns different weights to various domain scores and combinations of scores. The resulting score can then be used to determine the priority group. Initially, there were three proposed priority groups; the highest priority group includes scores 3–8, the intermediate priority is 9–12, and the lowest priority group consists of scores greater than 12. The score and its components are summarized in Supplemental Tables 1−4.

### 2.3. Statistical Analysis

Analysis was conducted using Microsoft Excel (Microsoft, Redmond WA) and R (R Core Team, 2018). Basic descriptive characteristics were analyzed and reported as mean with standard deviation or median with interquartile range. Two-tailed *t*-tests, Wilcoxon rank-sum test, and chi-square tests were used to determine if there were significant differences between the COVID-19 and non-COVID-19 groups. Using three regression models, we assessed whether increasing MN scores were associated with increased mortality, duration of ECMO cannulation, and discharge disposition. For ECMO duration, we performed a linear regression after log-transforming the variable, then assessed whether the regression was affected by COVID-19 status by adding it into the model as a covariate. This analysis was repeated for the RESP score. Using ROC analysis, statistically optimal cut points for the MN Score were identified for three levels of priority by maximizing sensitivity and specificity for in-hospital mortality. A chi-square test was then used to examine whether mortality was different between the priority levels.

## 3. Results

### 3.1. Patient Characteristics

A total of 124 patients met our inclusion criteria and were analyzed; their baseline characteristics, hospital disposition, and mortality are detailed in Table 1. The most prevalent comorbidities were obesity (55%), tobacco use (42%), hypertension (34%), diabetes (24%), and hyperlipidemia (23%). The mean BMI was 31 kg/m^2^ (SD 7). The mean SOFA score was 8 (SD 3), the mean RESP score was 2.2 (SD 3.1), and the mean MN score was 8 (SD 4). The mean time on ECMO was 17 days (SD 15). The overall in-hospital mortality rate was 38%; of the remaining patients, 14% were discharged to home and 48% were discharged to a rehabilitation facility. At 30 days post discharge, no additional mortalities were identified; however, 13 patients were lost to follow-up.

### 3.2. COVID-19 and Non-COVID-19 Groups

Most of the patient characteristics were not significantly different between the COVID-19 (*n* = 47) and non-COVID-19 groups (*n* = 77) (Table 1). The COVID-19 group had significantly older patients (median 53, IQR 46–57), *p*=0.006), a higher percentage of males (85%, *p*=0.014), more patients with diabetes mellitus (36%, *p*=0.02), fewer patients who use tobacco (17%, *p* < 0.001), and lower SOFA scores (mean 7, SD 2, *p* < 0.001). The distribution of race and ethnicity was significantly different between COVID-19 and non-COVID-19 groups (*p* < 0.001) with an increased frequency of self-identified Black (49%), Hispanic (34%), and Asian (11%) patients treated for COVID-19 ARDS.

### 3.3. Outcomes

The MN Score was significantly associated with in-hospital mortality (Table 2, OR = 1.13, 95% CI 1.02–1.24, *p*=0.02). Adjusting for the COVID-19 pneumonia ARDS as the primary indication for ECMO did not change the predictive ability of the MN score (OR = 1.43, 95% CI 0.64–3.21). The RESP score was not significantly associated with mortality (Table 2, OR = 0.94, 95% CI 0.84–1.07, *p*=0.39). Neither score was significantly associated with duration of ECMO cannulation (MN score *p*=0.051, *R*^2^ = 0.031, coefficient = −1.51; RESP score *p*=0.843, *R*^2^ = 0.0003, coefficient = 0.13) or disposition at hospital discharge to a rehab facility (MN score coefficient = 0.03, *R*^2^ = 0.03, *p*=0.74, RESP score coefficient = −0.13, *R*^2^ = 0.01, *p*=0.25).

### 3.4. Priority Groups

**Figure 2:**
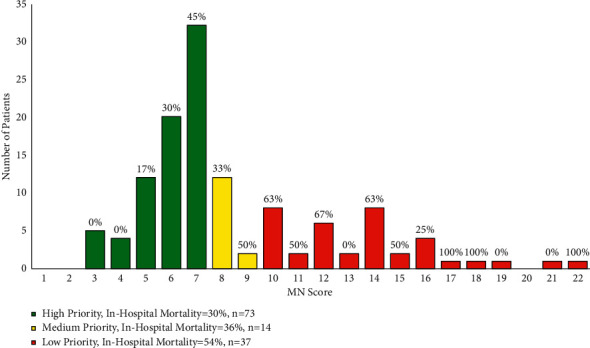
Minnesota Score distribution and priority groups that are statistically optimized for specificity and sensitivity. Percentages above each bar represents the mortality for that specific score. Chi-square analysis confirmed statistically significant increase in mortality between priority groups (*p*=0.05). ROC analysis showed an AUC of 0.66 for the first cut point and 0.55 for the second.

For the MN Score, statistically determined optimal cut points were ≤7 for high priority and >9 for low priority using in-hospital mortality as the metric to determine cut points. In-hospital mortality rates were calculated and found to be 30% for the high-priority group, 36% for the intermediate-priority group, and 54% for the low-priority group (Figure 2, *p*=0.05). ROC analysis showed an AUC of 0.66 (sensitivity 0.83, specificity 0.43, CI 6–10) for the first cut point and 0.55 (sensitivity 0.84, specificity 0.31, CI 9–22) for the second (Supplemental Figure 1). Supplemental Figure 4 shows these groups in COVID-19-positive patients only.

**Figure 3:**
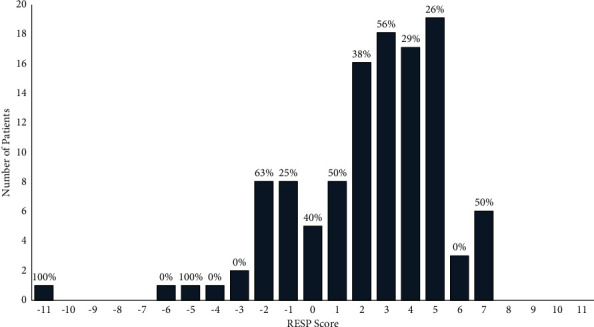
Respiratory ECMO survival prediction (RESP) score distribution and in-hospital mortality rates. There is no consistent pattern of increasing mortality with decreased RESP score.

We also tested a more extreme version of the priority groups that put the high priority group cut point at 4 since no patients with a score of 4 or lower died; the low priority group consisted of those with scores of 9 or higher. In-hospital mortality rates were calculated and found to be 0% for the high-priority group, 34% for the medium-priority group, and 54% for the low-priority group (Supplemental Figure 2, *p*=0.04). We also examined the originally proposed priority groups which had the cut points as 8 and 12. In-hospital mortality rates were calculated and found to be 31% for the high-priority group, 61% for the medium-priority group, and 48% for the low-priority group (Supplemental Figure 3, *p*=0.03). The distribution of the RESP score is shown in Figure 3 along with mortality rates for each score. Notably, survival rates do not appear to relate to increasing or decreasing RESP score.

## 4. Discussion

The COVID-19 pandemic led to a shortage of resources during critical periods of surging caseloads [8]. As a result, clinicians and healthcare systems have sought equitable and accurate systems of allocation for various resources, most prominently ventilators [15]. ECMO is a resource-intensive therapy, and multiple allocation algorithms have been proposed during the COVID-19 pandemic but given the short timeframe, none have been validated [12]. We evaluated the MN Score and found that it was significantly associated with in-hospital mortality and with further validation, could be used for allocation during resource scarcity.

The MN Score was developed by the Minnesota ECMO consortium, which consists of representatives from all four adult Extracorporeal Life Support Centers of Excellence in Minnesota. It is specific to ECMO and can be used for V-V or V-A patients [12]. It was inspired by a model developed at the University of Pittsburgh Medical Center for the ethical allocation of scarce critical care resources, particularly ventilators [13]. This score utilizes age, SOFA score, and underlying pathology which provides determines anticipated survival and duration of ECMO cannulation. However, this score was developed in a qualitative manner at the beginning of the pandemic when little to no data was available for COVID-19 patient outcomes on ECMO. With this study, we sought to statistically evaluate the MN Score by determining its associations with key outcomes and evaluating the accuracy of the cut points.

Our study found the MN Score was significantly associated with in-hospital mortality in patients treated with V-V ECMO for ARDS. COVID-19 status did not significantly change the result, suggesting that this model can be used in COVID-19 and non-COVID-19 patients, which is advantageous given the current uncertainty around the duration of the pandemic and its impacts on the healthcare system. The MN Score was not associated with discharge disposition or time on ECMO. This was surprising given the incorporation of expected ECMO duration as part of the MN score tier system. This suggests it may be difficult for clinicians to predict ECMO length and a score that more accurately predicts ECMO duration may be particularly useful during times of scarcity. In future studies, we hope to further evaluate the priority groups and their cut points. The middle group in particular is likely skewed by a low sample size and may not be clinically useful. We may find that with a larger data sample new cut points emerge, or that it would make more sense to have just a low- and high-priority category with no middle category.

Interestingly, RESP scores were not associated with in-hospital mortality in this cohort. Furthermore, the mortality rates for each RESP score did not show a consistent trend in our population (Figure 3). Given the study design and limited sample size, we cannot fully assess the use of the RESP score in allocation of scarce resources. However, the RESP score does have several shortcomings in the current environment while the MN score has several advantages. First, the MN score is easy to use and has relatively few components which are typically readily available for patients transferred from rural facilities or from out of state. In contrast, the RESP score requires extensive data which may be difficult to find or unavailable in many patients, especially those who are transferred from outside facilities [10, 16]. Particularly in the United States, where there is no national healthcare system and patients are often transferred to tertiary care centers, heavy reliance on specific labs or interventions can be a barrier to utility. In our study, over 60% of the patients were referred from a non-ECMO hospital, with ECMO frequently initiated prior to transfer. Second, the MN Score does not use comorbidities. From an equity perspective, comorbidities are increased in certain populations, and their use in determining benefit or allocation could exacerbate already existing health disparities [17]. This also increases the convenience of the score, as comorbidities are not always available for transfer patients or may not be apparent in patients who lack regular access to medical care. Finally, the MN Score lends itself to categorization in priority groups which can be used in the event of resource scarcity. While the originally proposed groups were not associated with monotonically increasing mortality, the statistically optimized cut points did show a monotonic increase in mortality.

There are limitations to our study as a preliminary statistical evaluation of the MN Score. First, the data analysis was performed in a retrospective manner and is therefore limited by increased risk for misclassification bias and confounding. Additionally, our sample size was small, which may have limited our ability to detect a statistically significant effect with the RESP score. This small sample size also limited our ability to analyze individual elements of the Minnesota Score and further optimize cut points. Another limitation was that the COVID-19 patients were from multiple sites, which may have had institutional differences and varied protocols that could have impacted the results. This was in part mitigated by the fact that all the sites were part of a consortium with agreed upon selection criteria and care protocols. Similarly, most of the non-COVID-19 patients were from prior to 2020. Therefore, it is possible that substantial secular trends in critical care management of ARDS or ECMO may have contributed to our findings. The groups also contained some statistically significant differences, including different age, sex, race, and rates of diabetes mellitus. These differences can likely be attributed to underlying risk factors for severe COVID-19 ARDS, which have been well established [18]. Finally, our study only examines the scores in the context of ARDS treated with V-V ECMO. These results are not generalizable to V-A ECMO patients or V-V ECMO patients with other indications for ECMO, including bridge to transplant. Nevertheless, the MN Score does have internal and face validity within the context of COVID-19 and is at a minimum appropriate for this population. To address some of these limitations, we believe the MN Score should be further validated in larger populations with split training and testing datasets and eventually incorporated into a prospective trial. One mechanism for increasing sample size is using the ELSO registry, and we hope to use this data in future studies to further evaluate the MN Score.

## 5. Conclusions

The MN Score is associated with increased mortality and proposed priority groups can be utilized for allocation of ECMO in times of increasing scarcity.

## Figures and Tables

**Table 1:** Demographics and comorbidities of patients treated with venovenous extracorporeal membrane oxygenation for acute respiratory distress syndrome with and without COVID-19.

Characteristic		All patients *n* = 124	Without COVID-19 *n* = 77	With COVID-19 *n* = 47	*p* value
Age, median (IQR)		48 (38–57)	43 (34–57)	53 (46–57)	0.006^*∗*^
Sex, *n* (%)	Male	91 (73)	51 (66)	40 (85)	0.014^*∗*^

Race, *n* (%)	American Indian	3 (2)	2 (3)	1 (2)	<0.001^*∗*^
	Asian	8 (6)	3 (4)	5 (11)	
	Black	21 (17)	10 (13)	11 (49)	
	White, Hispanic	20 (16)	4 (5)	16 (34)	
	White, non-Hispanic	66 (53)	52 (68)	14 (30)	
BMI, mean (SD)		31 (7)	31 (7)	32 (6)	0.86

Comorbidity, *n* (%)	Obesity	68 (55)	42 (55)	26 (55)	0.93
	Tobacco use	52 (42)	44 (57)	8 (17)	<0.001^*∗*^
	Hypertension	42 (34)	23 (30)	19 (40)	0.22
	Diabetes mellitus	30 (24)	13 (17)	17 (36)	0.02^*∗*^
	Hyperlipidemia	28 (23)	16 (21)	12 (26)	0.54
	Asthma	13 (10)	10 (13)	3 (6)	0.24
	COPD	12 (10)	10 (13)	2 (4)	0.11
	Coronary artery disease	10 (8)	7 (9)	3 (6)	0.59
	Chronic kidney disease	9 (7)	5 (6)	4 (9)	0.67
SOFA, mean (SD)		8 (3)	9 (3)	7 (2)	<0.001^*∗*^
RESP, mean (SD)		2.2 (3.1)	2.2 (3.0)	2.2 (3.3)	0.98

Disposition, *n* (%)	Home	15 (14)	12 (17)	3 (7)	0.12
	Rehabilitation	48 (43)	31 (45)	17 (40)	0.65
In-hospital mortality, *n* (%)		47 (38)	29 (38)	18 (38)	0.94

SOFA, sequential organ failure assessment score; RESP, respiratory ECMO survival prediction score; BMI, body mass index; COPD, chronic obstructive pulmonary disease; COVID-19, severe acute respiratory syndrome coronavirus 2. ^*∗*^*p* < 0.05.

**Table 2:** In-hospital mortality odds ratios for the Minnesota Score and respiratory ECMO survival prediction score. For one point increase in the score, the odds ratio represents an increase in mortality.

Scoring system	In-hospital mortality odds ratio (95% CI, *p* value)
MN	1.13 (1.02–1.24, 0.02^*∗*^)
RESP	0.94 (0.84–1.07, 0.39)

MN, Minnesota ECMO score; RESP, respiratory ECMO survival prediction score; CI, confidence interval. ^*∗*^*p* value <0.05.

## Data Availability

The data are not available for public use.

## Abbreviations

ECMO: Extracorporeal membrane oxygenation
V-V-: Venovenous
V-A-: Venoarterial
ARDS: Acute respiratory distress syndrome
COVID-19: SARS-CoV-2 virus
ELSO: Extracorporeal life support organization
RESP: Respiratory ECMO survival prediction score
MN: Minnesota
SOFA: Sequential organ failure assessment score.

## Supplementary Materials

Supplementary Materials Supplemental Figure 1: ROC curves for the two cut points used to create priority groups for allocation of V-V ECMO during scarcity. ROC analysis showed an AUC of 0.66 for the first cut point and 0.55 for the second. Supplemental Figure 2: Minnesota Score distribution using more extreme priority groups where no one died in the high priority group. Chi-square analysis confirmed statistically significant increase in mortality between priority groups (*p*=0.04). Supplemental Figure 3: Minnesota Score distribution using the originally proposed priority groups. There is not a consistent increase in mortality between the groups. Supplemental Figure 4: Minnesota Score distribution and statistically optimized priority groups in COVID-19-positive patients only. There is not a clear pattern due to small sample size in the middle-priority group (*n* = 1). Supplemental Table 1: Minnesota Score predicted survival and duration of ECMO. Supplemental Table 2: Minnesota Score anticipated survival and duration matrix. Supplemental Table 3: Minnesota Score Total Points for Priority Score (range of possible scores 3–22). Supplemental Table 4: Minnesota Score priority groups as originally proposed, statistically optimized, and severe.

## References

[1] Munshi L., Walkey A., Goligher E., Pham T., Uleryk E. M., Fan E. (2019). Venovenous extracorporeal membrane oxygenation for acute respiratory distress syndrome: a systematic review and meta-analysis. *The Lancet Respiratory Medicine*.

[2] Huang C., Wang Y., Li X. (2020). Clinical features of patients infected with 2019 novel coronavirus in Wuhan, China. *The Lancet*.

[3] Marini J. J., Gattinoni L. (2020). Management of COVID-19 respiratory distress. *JAMA*.

[4] Chen N., Zhou M., Dong X. (2020). Epidemiological and clinical characteristics of 99 cases of 2019 novel coronavirus pneumonia in Wuhan, China: a descriptive study. *The Lancet*.

[5] Barbaro R. P., MacLaren G., Boonstra P. S. (2020). Extracorporeal membrane oxygenation support in COVID-19: an international cohort study of the extracorporeal life support organization registry. *Lancet*.

[6] Bergman Z. R., Wothe J. K., Alwan F. S. (2021). The use of venovenous extracorporeal membrane oxygenation in COVID-19 infection: one region’s comprehensive experience. *ASAIO Journal*.

[7] Combes A., Hajage D., Capellier G. (2018). Extracorporeal membrane oxygenation for severe acute respiratory distress syndrome. *New England Journal of Medicine*.

[8] Ranney M. L., Griffeth V., Jha A. K. (2020). Critical supply shortages - the need for ventilators and personal protective equipment during the covid-19 pandemic. *New England Journal of Medicine*.

[9] Ramanathan K., Antognini D., Combes A. (2020). Planning and provision of ECMO services for severe ARDS during the COVID-19 pandemic and other outbreaks of emerging infectious diseases. *The Lancet Respiratory Medicine*.

[10] Schmidt M., Bailey M., Sheldrake J. (2014). Predicting survival after extracorporeal membrane oxygenation for severe acute respiratory failure. The respiratory extracorporeal membrane oxygenation survival prediction (RESP) score. *American Journal of Respiratory and Critical Care Medicine*.

[11] Bartlett R. H., Ogino M. T., Brodie D. (2020). Initial ELSO guidance document: ECMO for COVID-19 patients with severe cardiopulmonary failure. *ASAIO Journal*.

[12] Prekker M. E., Brunsvold M. E., Bohman J. K. (2020). Regional planning for extracorporeal membrane oxygenation allocation during coronavirus disease 2019. *Chest*.

[13] White D. B., Lo B. (2020). A framework for rationing ventilators and critical care beds during the COVID-19 pandemic. *JAMA*.

[14] Harris P. A., Taylor R., Thielke R., Payne J., Gonzalez N., Conde J. G. (2009). Research electronic data capture (REDCap)-A metadata-driven methodology and workflow process for providing translational research informatics support. *Journal of Biomedical Informatics*.

[15] Dos Santos M. J., Martins M. S., Santana F. L. P. (2020). COVID-19: instruments for the allocation of mechanical ventilators-a narrative review. *Critical Care*.

[16] Senussi M. H. (2014). Respite from the RESP score. *American Journal of Respiratory and Critical Care Medicine*.

[17] Röösli E., Rice B., Hernandez-Boussard T. (2021). Bias at warp speed: how AI may contribute to the disparities gap in the time of COVID-19. *Journal of the American Medical Informatics Association*.

[18] Zhou F., Yu T., Du R. (2020). Clinical course and risk factors for mortality of adult inpatients with COVID-19 in Wuhan, China: a retrospective cohort study. *The Lancet*.

