# Supplementary material for: Evaluation of Minnesota Score in the Allocation of Venovenous Extracorporeal Membrane Oxygenation During Resource Scarcity
Source: Crit Care Res Pract. 2022 Apr 6;2022:2773980. doi: 10.1155/2022/2773980 (PMC8985705; doi:10.1155/2022/2773980)
Supplement: Supplementary Materials — Supplemental Figure 1: ROC curves for the two cut points used to create priority groups for allocation of V-V ECMO during scarcity. ROC analysis showed an AUC of 0.66 for the first cut point and 0.55 for the second. Supplemental Figure 2: Minnesota Score distribution using more extreme priority groups where no one died in the high priority group. Chi-square analysis confirmed statistically significant increase in mortality between priority groups (p=0.04). Supplemental Figure 3: Minnesota Score distribution using the originally proposed priority groups. There is not a consistent increase in mortality between the groups. Supplemental Figure 4: Minnesota Score distribution and statistically optimized priority groups in COVID-19-positive patients only. There is not a clear pattern due to small sample size in the middle-priority group (n = 1). Supplemental Table 1: Minnesota Score predicted survival and duration of ECMO. Supplemental Table 2: Minnesota Score anticipated survival and duration matrix. Supplemental Table 3: Minnesota Score Total Points for Priority Score (range of possible scores 3–22). Supplemental Table 4: Minnesota Score priority groups as originally proposed, statistically optimized, and severe. [file 2773980.f1.zip › 2773980.f1/Supplemental Tables.docx]

Supplemental Table 1. Minnesota Score Predicted Survival and Duration of ECMO

| **Predicted Survival** | **Short Duration (≤5 days)** | **Long Duration ECMO (>5 days)** |
| --- | --- | --- |
| **Tier 1 (>60%)** | Acute hypercarbic respiratory failure because of status asthmaticus | Acute respiratory failure because of infection (especially influenza or coronavirus) with single organ failure |
|  | Cardiac arrest or cardiogenic shock because of severe accidental hypothermia | Acute respiratory failure because of trauma (drowning, pulmonary contusion, etc) with single organ failure |
|  | Pediatric pre- and post-cardiotomy cardiogenic shock | Pediatric myocarditis |
|  | Neonatal meconium aspiration syndrome | Other neonatal indications (including sepsis, congenital diaphragmatic hernia, and persistent pulmonary hypertension of the newborn) |
| **Tier 2 (30-60%)** | Poisoning-induced cardiogenic shock | Acute respiratory failure from any cause with multiorgan failure (including kidney injury requiring dialysis or hypotension requiring vasopressor support) |
|  | Massive pulmonary embolism | Pediatric/neonatal cardiac arrest from a cardiac etiology |
| **Tier 3 (<30%)** | Adult post-cardiotomy cardiogenic shock | Bridge to lung transplantation for irreversible respiratory failure |
|  | Out-of-hospital, refractory cardiac arrest with favorable prognostic features (i.e., extracorporeal CPR) | Acute respiratory failure and severe immunocompromise (e.g., stem cell transplant < 240 days posttransplant) |
|  | Cardiac arrest with non-shockable rhythm or unfavorable prognostic features (including most adults with in-hospital cardiac arrest) | Cardiovascular collapse refractory to vasopressors in the setting of multiorgan failure of any cause (e.g., septic shock) |

Supplementary Table 2. Minnesota Score Anticipated Survival and Duration Matrix

|  | **Duration** | |
| --- | --- | --- |
| **Anticipated Survival** | **Short (<5 days)** | **Long (≥5 days)** |
| **>60%** | A | B |
| **30-60%** | C | D |
| **<30%** | E | F |

Supplemental Table 3. Minnesota Score Total Points for Priority Score (range of possible scores 3-22).

| **Points** | **1** | **2** | **3** | **4** | **5** | **6** | **7** | **8** | **9** | **10** | **11** | **12** |
| --- | --- | --- | --- | --- | --- | --- | --- | --- | --- | --- | --- | --- |
| **Matrix** | A | B |  |  |  | C | D |  |  |  | E | F |
| **SOFA** | <6 | 6-8 | 9-11 | ≥12 |  |  |  |  |  |  |  |  |
| **Age** | <40 |  | 41-60 |  |  | 61-75 |  |  |  |  |  |  |

Supplemental Table 4. Minnesota Score priority groups as originally proposed, statistically optimized, and severe.

| Priority Group | Originally Proposed | Statistically Optimized | Severe |
| --- | --- | --- | --- |
| GREEN  Highest Priority | Score 3-8 | Score 3-7 | Score 3-4 |
| YELLOW  Intermediate Priority | Score 9-12 | Score 8-9 | Score 5-9 |
| RED  Lowest Priority | Score ≥13 | Score >9 | Score >9 |
