# Supplementary figures and images for: Evaluation of Minnesota Score in the Allocation of Venovenous Extracorporeal Membrane Oxygenation During Resource Scarcity
Source: Crit Care Res Pract. 2022 Apr 6;2022:2773980. doi: 10.1155/2022/2773980 (PMC8985705; doi:10.1155/2022/2773980)

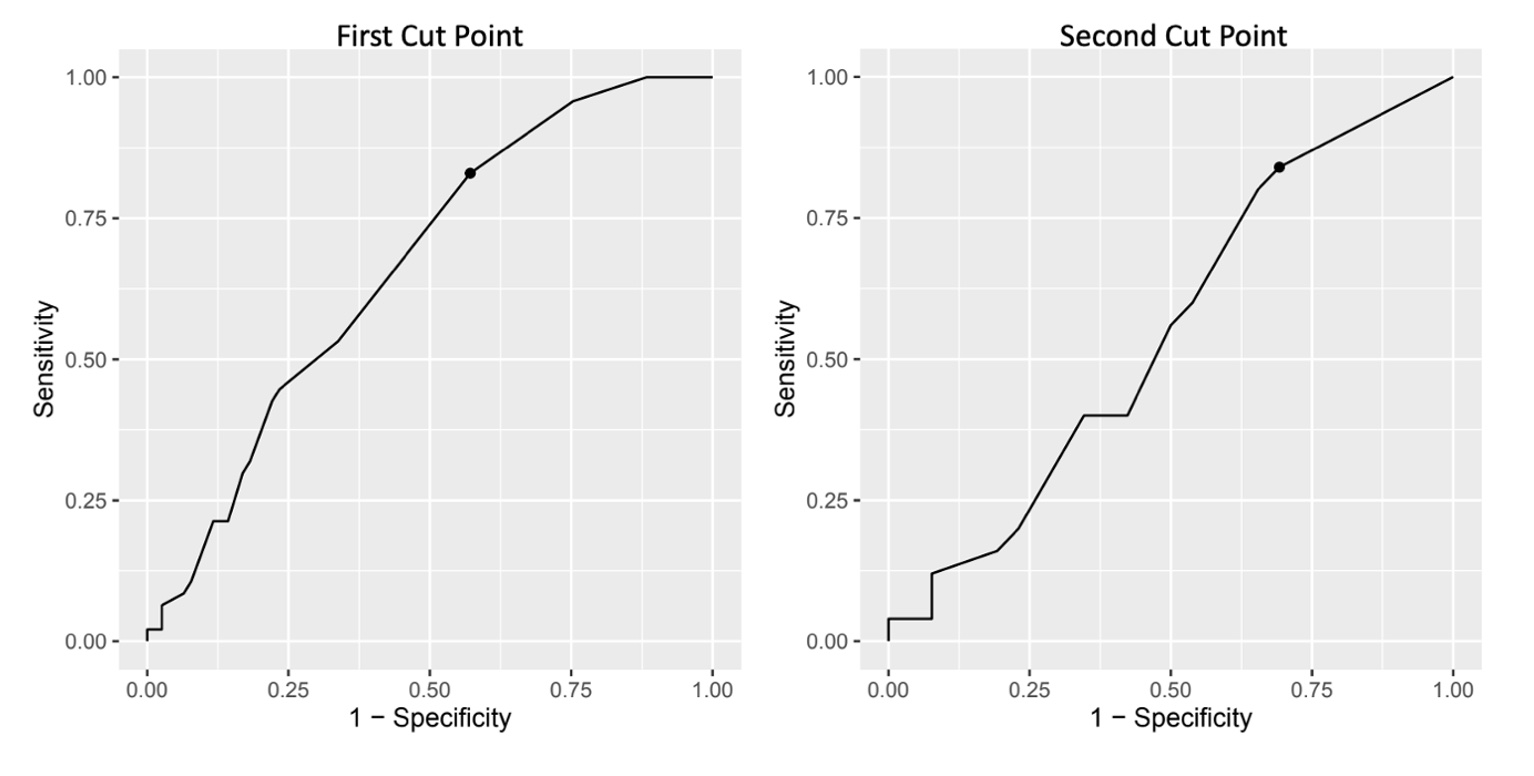

Supplement: Supplementary Materials — Supplemental Figure 1: ROC curves for the two cut points used to create priority groups for allocation of V-V ECMO during scarcity. ROC analysis showed an AUC of 0.66 for the first cut point and 0.55 for the second. Supplemental Figure 2: Minnesota Score distribution using more extreme priority groups where no one died in the high priority group. Chi-square analysis confirmed statistically significant increase in mortality between priority groups (p=0.04). Supplemental Figure 3: Minnesota Score distribution using the originally proposed priority groups. There is not a consistent increase in mortality between the groups. Supplemental Figure 4: Minnesota Score distribution and statistically optimized priority groups in COVID-19-positive patients only. There is not a clear pattern due to small sample size in the middle-priority group (n = 1). Supplemental Table 1: Minnesota Score predicted survival and duration of ECMO. Supplemental Table 2: Minnesota Score anticipated survival and duration matrix. Supplemental Table 3: Minnesota Score Total Points for Priority Score (range of possible scores 3–22). Supplemental Table 4: Minnesota Score priority groups as originally proposed, statistically optimized, and severe. [file 2773980.f1.zip › 2773980.f1/MN Score Supplemental Figure 1.jpg]

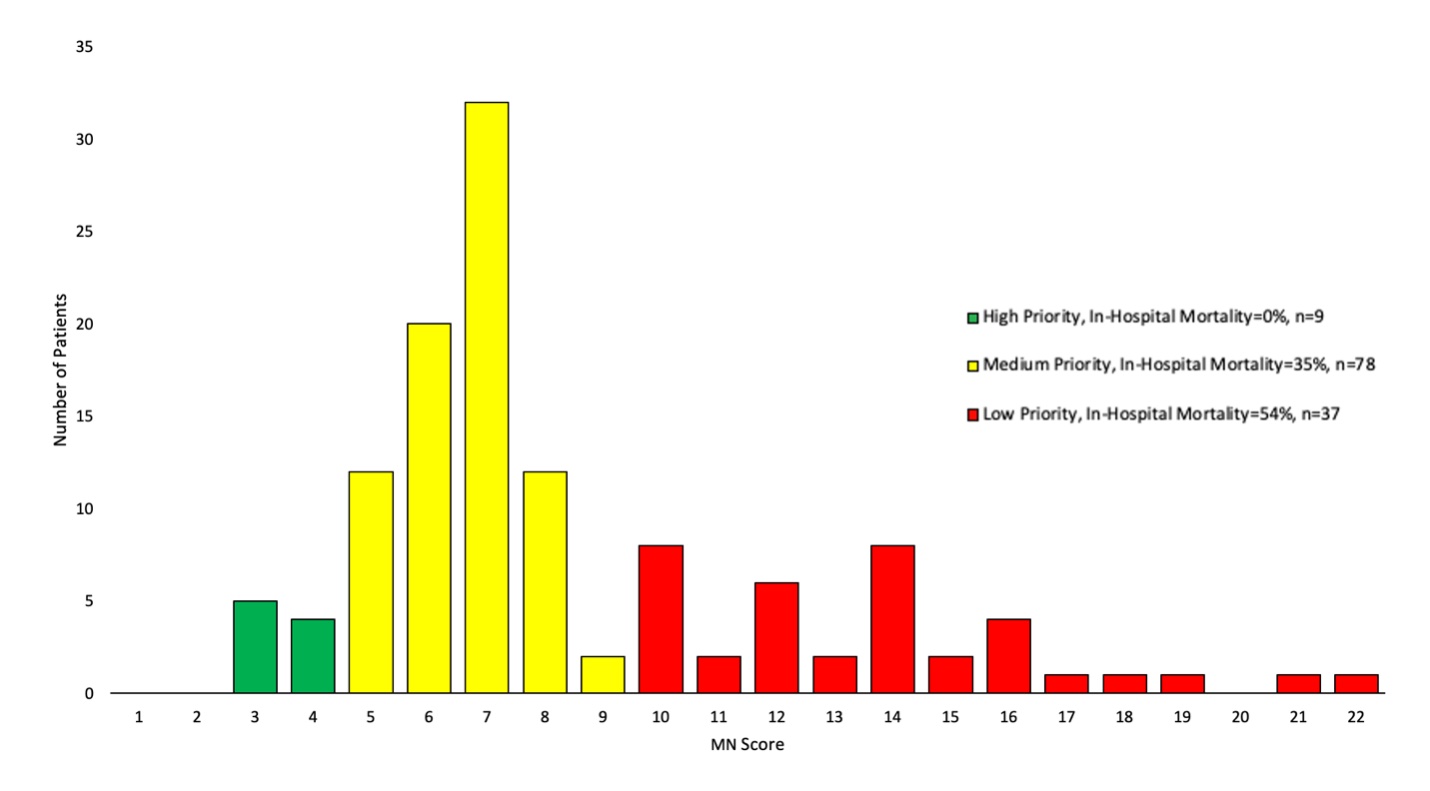

Supplement: Supplementary Materials — Supplemental Figure 1: ROC curves for the two cut points used to create priority groups for allocation of V-V ECMO during scarcity. ROC analysis showed an AUC of 0.66 for the first cut point and 0.55 for the second. Supplemental Figure 2: Minnesota Score distribution using more extreme priority groups where no one died in the high priority group. Chi-square analysis confirmed statistically significant increase in mortality between priority groups (p=0.04). Supplemental Figure 3: Minnesota Score distribution using the originally proposed priority groups. There is not a consistent increase in mortality between the groups. Supplemental Figure 4: Minnesota Score distribution and statistically optimized priority groups in COVID-19-positive patients only. There is not a clear pattern due to small sample size in the middle-priority group (n = 1). Supplemental Table 1: Minnesota Score predicted survival and duration of ECMO. Supplemental Table 2: Minnesota Score anticipated survival and duration matrix. Supplemental Table 3: Minnesota Score Total Points for Priority Score (range of possible scores 3–22). Supplemental Table 4: Minnesota Score priority groups as originally proposed, statistically optimized, and severe. [file 2773980.f1.zip › 2773980.f1/MN Score Supplemental Figure 2.jpg]

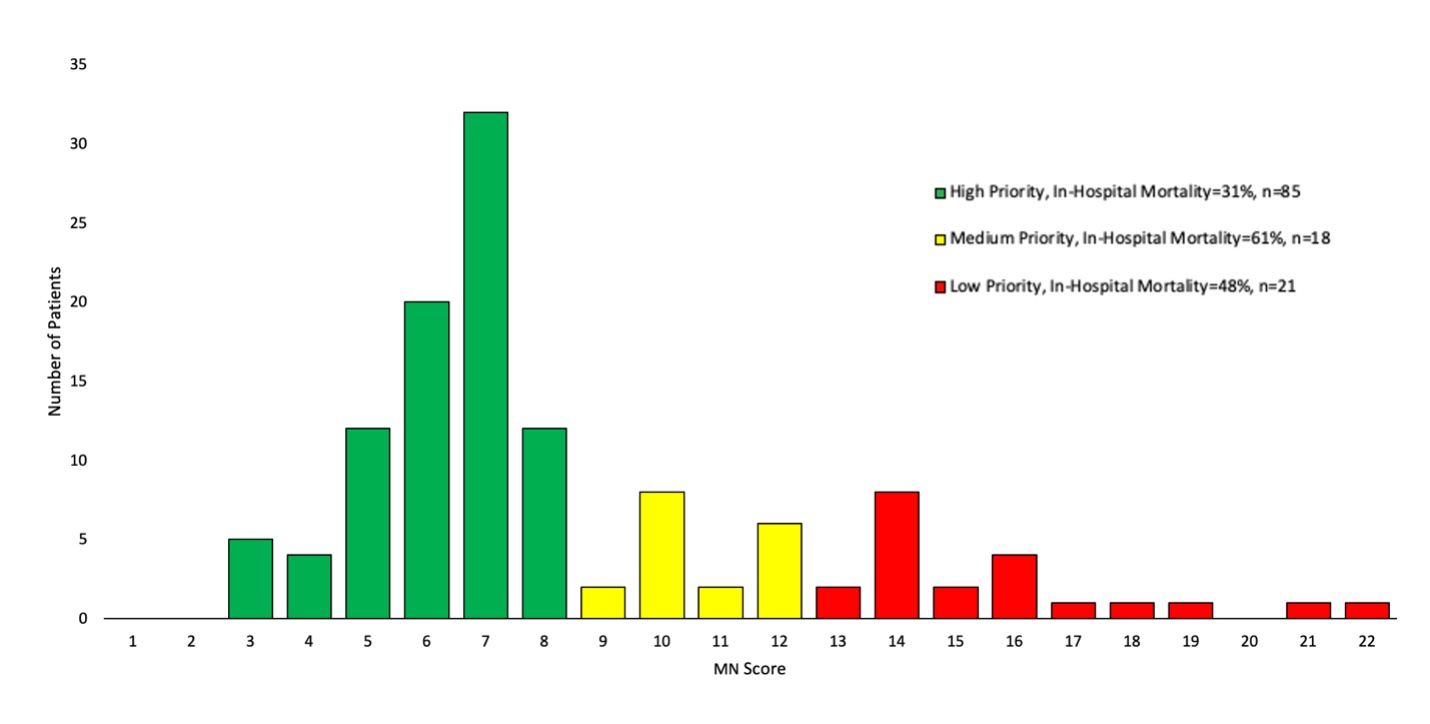

Supplement: Supplementary Materials — Supplemental Figure 1: ROC curves for the two cut points used to create priority groups for allocation of V-V ECMO during scarcity. ROC analysis showed an AUC of 0.66 for the first cut point and 0.55 for the second. Supplemental Figure 2: Minnesota Score distribution using more extreme priority groups where no one died in the high priority group. Chi-square analysis confirmed statistically significant increase in mortality between priority groups (p=0.04). Supplemental Figure 3: Minnesota Score distribution using the originally proposed priority groups. There is not a consistent increase in mortality between the groups. Supplemental Figure 4: Minnesota Score distribution and statistically optimized priority groups in COVID-19-positive patients only. There is not a clear pattern due to small sample size in the middle-priority group (n = 1). Supplemental Table 1: Minnesota Score predicted survival and duration of ECMO. Supplemental Table 2: Minnesota Score anticipated survival and duration matrix. Supplemental Table 3: Minnesota Score Total Points for Priority Score (range of possible scores 3–22). Supplemental Table 4: Minnesota Score priority groups as originally proposed, statistically optimized, and severe. [file 2773980.f1.zip › 2773980.f1/MN Score Supplemental Figure 3.jpg]

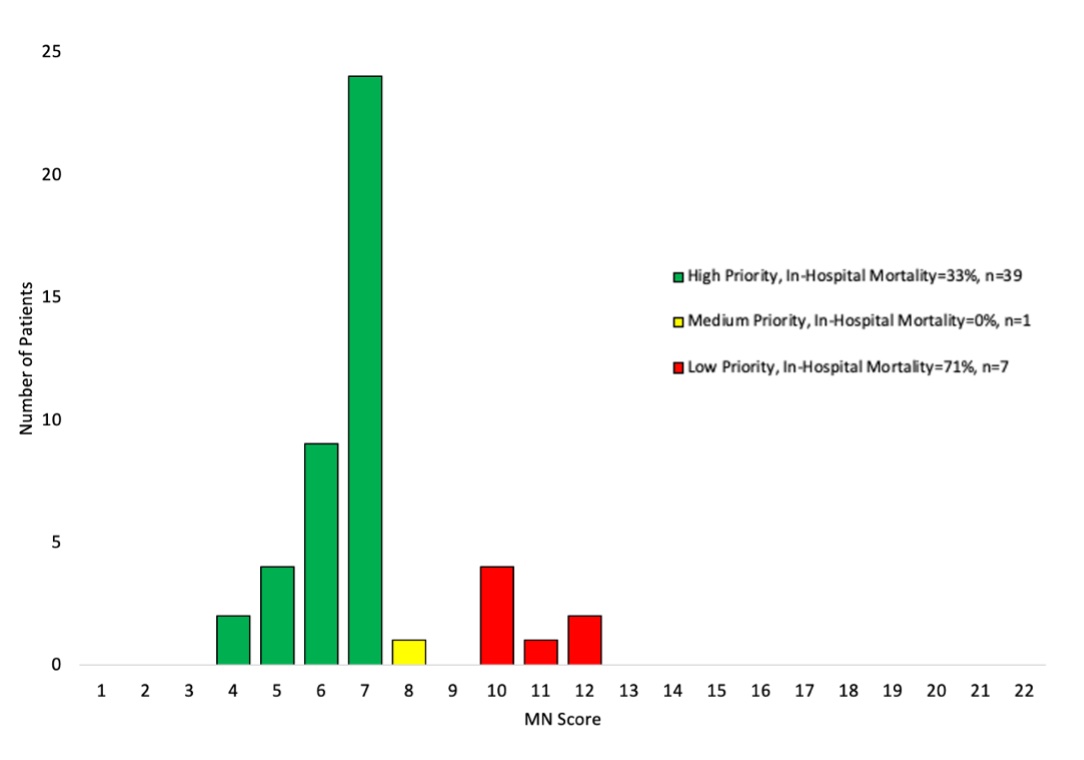

Supplement: Supplementary Materials — Supplemental Figure 1: ROC curves for the two cut points used to create priority groups for allocation of V-V ECMO during scarcity. ROC analysis showed an AUC of 0.66 for the first cut point and 0.55 for the second. Supplemental Figure 2: Minnesota Score distribution using more extreme priority groups where no one died in the high priority group. Chi-square analysis confirmed statistically significant increase in mortality between priority groups (p=0.04). Supplemental Figure 3: Minnesota Score distribution using the originally proposed priority groups. There is not a consistent increase in mortality between the groups. Supplemental Figure 4: Minnesota Score distribution and statistically optimized priority groups in COVID-19-positive patients only. There is not a clear pattern due to small sample size in the middle-priority group (n = 1). Supplemental Table 1: Minnesota Score predicted survival and duration of ECMO. Supplemental Table 2: Minnesota Score anticipated survival and duration matrix. Supplemental Table 3: Minnesota Score Total Points for Priority Score (range of possible scores 3–22). Supplemental Table 4: Minnesota Score priority groups as originally proposed, statistically optimized, and severe. [file 2773980.f1.zip › 2773980.f1/MN Score Supplemental Figure 4.jpg]
